# Supplementary material for: Increased Local Testosterone Levels Alter Human Fallopian Tube mRNA Profile and Signaling
Source: Cancers (Basel). 2023 Mar 30;15(7):2062. doi: 10.3390/cancers15072062 (PMC10093055; doi:10.3390/cancers15072062)
Supplement: Supplementary file 1 [file cancers-15-02062-s001.zip › cancers-2219948-supplementary.pdf]

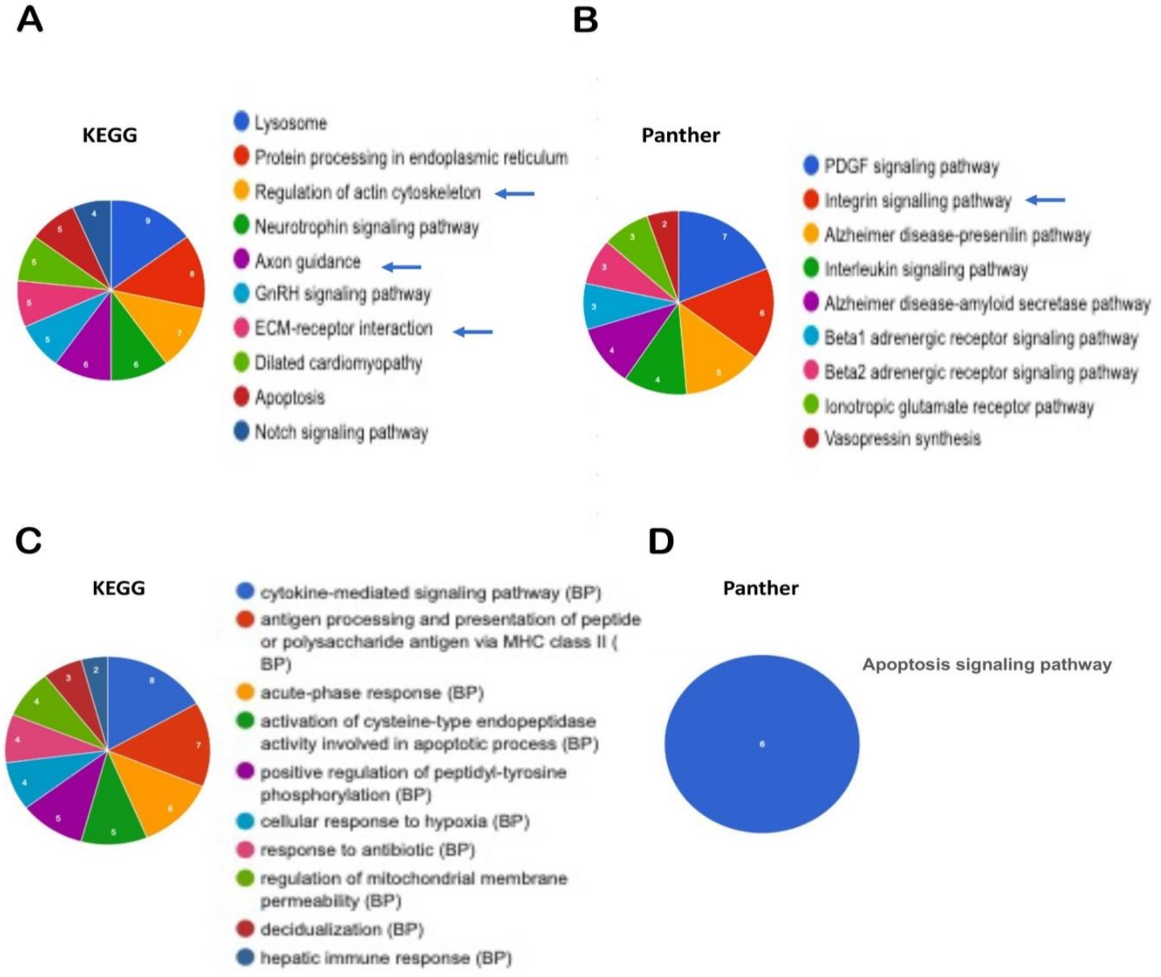

Figure S1. Pathway analysis of genes regulated by testosterone. A-B) Upregulated genes. B-C) Downregulated genes.

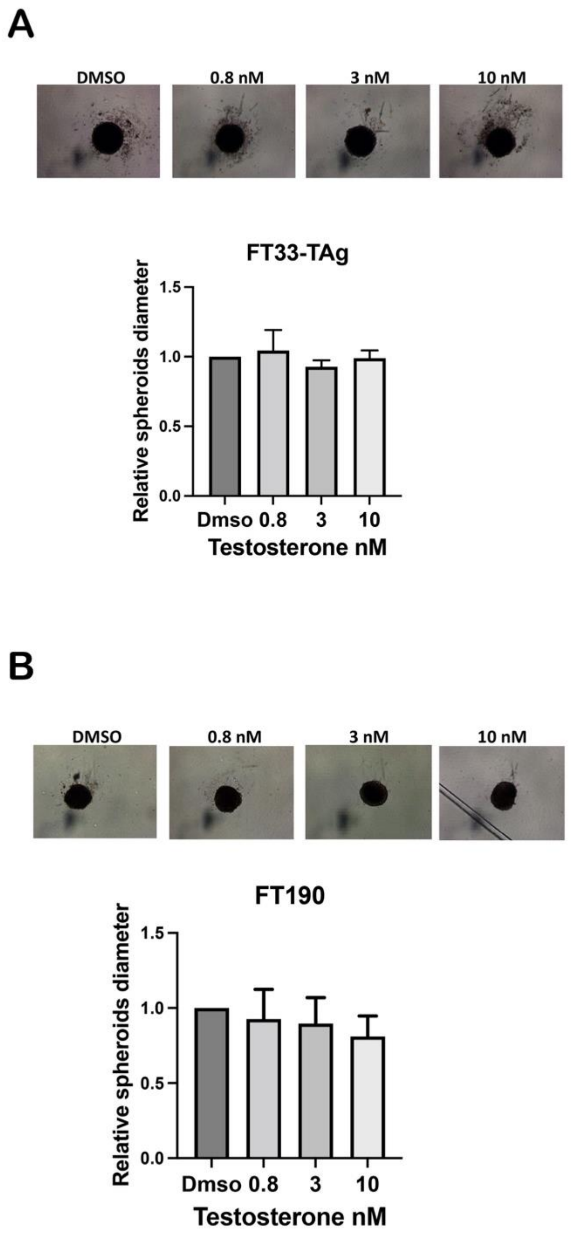

Figure S2. Effect of testosterone on spheroids size. A) FT33-TAg cells were seeded in ULA plates and grown for 10 days before taking pictures. Three independent experiments were performed and quantified using Image J to measure the diameter. One-Way Anova was performed with no significant differences. B) Same as A conducted in FT190 cells.

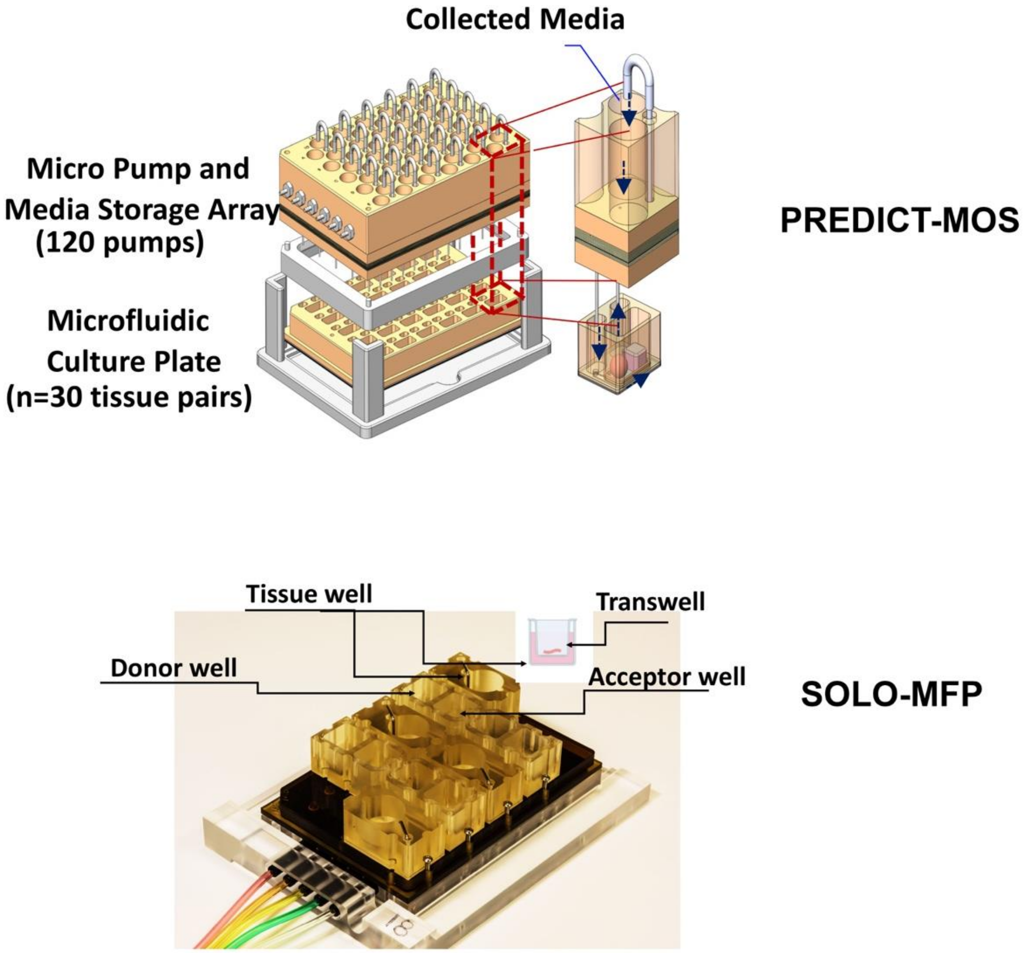

Figure S3. PTEDICT-MOS vs. SOLO microfluidic platform. Schematic of the PREDICT-MOS and the SOLO-MFP systems. Top panel shows the novel PREDICT system with the 30-tissue-well format. Bottom panel shows the older system SOLO-MFP with limited number of tissue-wells (about 4).

Table S1

Primers	forward	reverse
PIKB	ATCCAGAGTTCAGCTGCCA	CTCTTTTGCATCTTCCTTCAC
STEAP4v1	GCTTGGTAGCTCTGGGATTT	TTGCCTGGGTAACGGTTAAG
STEAP4v3	TCCAGTCCAAACTGGGTTATTT	GATTTGAAGGGCTGAGGAATCT
ZBTB16	TGTTTGAGATCCTCTTCCACCGC	TCTCCAGCATCTTCAGGCACTGT
KIF5C	GCCGAATGCAGCATCAAA	TTCCCTTGCCCGATCAC
TP53	AGCCAAGTCTGTGACTTGCA	AACCTCCGTCATGTGCTGT
P21	AAATCGTCCAGCGACCTTCCTCAT	TCTGACTCCTTGTTCCGCTGCTAA
CD82	ATCTCTGTCCTGCAAACCTCCTCC	GAAGCCCATGAGCATAGTG
PAX2	TGTGTCAGCAAAATCCTGG	CGGGTTCTGTCGTTTGTATTC
LAMA2	AGTCCTCAGGTGGAAGATAG	ACATGACAGTGGAGATGTTG
LGR6	ACGGCTTACCTGGACCTCA	GCTTGTCCTGGGATGTGTGA
WNT4	GAGCAACTGGCTGTACCTGG	TTCTCCTTCAGTGCGTGACC
18S	GCTTGCGTTGATTAAGTCCC	GCCTCACTAAACCATCCAATC

Table S2

Antibodies	Company	Catalog#
LGR6	ProteinTech	17658-I-AP
WNT4	Abcam	ab91226
